# Hospital and Health News

**Published:** 1923-12

**Authors:** 


### HOSPITAL AND HEALTH NEWS.

### Royal Sanitary Institute Congress.

The next Congress of the Royal Sanitary Institute will be held at Liverpool from July 14th to 19th, 1924.

### Alton and East Ham.

Mr. W. B. Harper, Secretary of the Lord Mayor Treloar's Cripple Homes at Alton, has been elected Mayor of East Ham for the ensuing year.

### Hospital Reduces Its Debt.

At the Coventry and Warwickshire Hospital the overdraft has been reduced from £30,000 to £14,000 in the past twelve months.

### "I Bring Thee Violets."

On his recent visit to Winchester, the Prince of Wales presented one of the bunches of violets thrown into his car to Miss Carpenter Turner, the Matron of the Royal Hampshire County Hospital.

### Director of the Rockefeller School.

The Transitional Executive Committee, under the Chairmanship of the Minister of Health, has appointed Dr. Andrew Balfour to be Director of the School of Hygiene which is to be established as a result of the gift of the Rockefeller Foundation.

### Cats Carry Ringworm.

The Persian kitten was declared by Dr. Alistair Mackenzie, in a lecture he gave at Dundee recently, to be a sure carrier of ringworm. A skin specialist had said that in every Persian kitten he found ringworm.

### Royal Portsmouth Hospital.

The Royal Portsmouth Hospital, the income of which from contributors has suffered much from unemployment, has received £133 from two approved societies under the Insurance Act, and Mr. Wagstaff, the Secretary, records that in all £1,200 has been received from this quarter.

### Royal Sussex County Hospital.

All the wards at the Royal Sussex County Hospital have now been reopened. Mr. Harry Preston, the hospital's President, has secured in all £15,000 for the hospital's funds, and lately stated that his next effort would be even more ambitious.

### Unit System Unsatisfactory.

After three years' experience, the London Hospital has found that the unit system, whereby specialists are induced to abandon private practice and in return for a salary to devote themselves to teaching and research, is unsatisfactory. A teacher is found to lose touch with the wider question of a patient's welfare and to concentrate too much upon the "case." The unit system, therefore, is being abandoned.

### Preserved and Coloured Food.

The Minister of Health has appointed Mr. P. A. Ellis Richards, F.I.C., President of the Society of Public Analysts, to be a member of the Departmental Committee on Preservatives and Colouring Matters in Food in the place of Mr. Otto Hehner, F.I.C., who has resigned from the Committee in consequence of having to return to South Africa.

### One Hospital for Swindon.

A meeting was recently held at Swindon to consider its hospital needs, at which a resolution was carried to the effect that one general hospital for the town was desirable. Mr. Roden Orde, a summary of whose report on the situation we published in our October number, was among those present. The Mayor is choosing a committee, representative of all sections affected in the town, to discuss further details.

### An Ideal X-Ray Department.

Sir Humphrey Rolleston, P.R.C.P., has opened the new X-Ray Department at the Weymouth and District Hospital, which is also a memorial to the late Dr. James Miller. Dr. Rodier Heath, the medical officer in charge of the department, described the X-Ray Department at Manchester Royal Infirmary, which now occupies a large wing and has nine sets of apparatus, as the ideal that he had tried to follow in miniature.

### The Nobel Prizes for Medicine.

The Nobel Prize for Medicine for 1923 has been awarded to Dr. Banting and Professor Macleod, of Toronto, for the discovery of insulin. The Nobel Prize for Medicine for 1922 has been divided between Professor Archibald V. Hill, University College, London, and Professor Otto Meyerhof, Professor of Physiology at Kiel University.

### Appointments and Vacancies.

The appointment of Mr. H. K. Pearce to be Secretary of the South Devon and East Cornwall Hospital, Plymouth, has created a vacancy in the post of chief clerk at Manchester Royal Infirmary. The salary is £400 a year. Mr. John S. Birbeck is the new Secretary of the Salisbury Infirmary. First at the Royal Southern Hospital, Liverpool, Mr. Birbeck became Assistant Secretary at the Nottingham General Hospital.

### Twopence a Week.

The Friendly Societies of Bath are asking 12,000 of the inhabitants severally to contribute 2d. a week to the Royal United Hospital. Already 2,500 people have been enrolled, and Canterbury's achievement on the same lines is held up to the emulation of Bath. Major James, Chairman of the Kent and Canterbury Scheme, said that the Kent Scheme was working in 100 towns and villages, and that the Bath area contained 60,000 families. It would surely be better to ask all to join than to limit the appeal to 12,000.

### Christmas Number of "Truth."

The Christmas number of *Truth* is, as usual, an exceedingly entertaining production, well illustrated and full of varied matter. The chief item this year is a clever political skit, "The Adventures of Don Quack-shott de La Marxa ("Knight of the Horny Hand"), otherwise Mr. Ramsay MacDonald, and two coloured supplements are included in the number, which can be obtained for 1s. 6d.

### Cadbury's Chocolates.

From Messrs. Cadbury we have received some very attractive boxes of chocolates, including the well-known King George Chocolates and the Carnival Chocolates, which are perhaps the most tempting of all the delicious manufactures of this firm. The Bournville Chocolate is one of the best plain chocolates to be obtained, and there is also a Dairy Milk variety for those who prefer it. Half-pound packets of these cost 1s. 3d. A pure and nourishing cocoa is another of Messrs. Cadbury's productions, and, like all these goods, is manufactured under excellent conditions and is of the finest quality.

### Dominions Support Cancer Campaign.

Following the announcement that in New Zealand a Cancer Campaign has been started with great enthusiasm, the British Red Cross Society are informed that in Australia they are assured of full support—in both cases the fullest co-operation has been secured with the central body in London and subscriptions will go to swell the general fund. The Governor-General of Ceylon is recommending the Legislative Council to make an annual vote of Rs. 15,000, which amounts to £666.

### Valentine's Meat Juice.

Valentine's Meat Juice has long been recognised as an admirable form of nourishment, and both here and in the United States it is being increasingly used. Many medical men have found it valuable in cases when all other nutriment has failed, and during a recent typhoid epidemic a few of the most hopeless cases were kept alive for ten days on nothing but Valentine's Meat Juice and a little water. It is useful for the relief of nausea, and when cod-liver oil is objectionable to the palate, a little meat juice in it renders the combination acceptable.

### The Treloar Cripples' Hospital.

In his will the late Sir William Treloar expressed the desire that Sir William Henry Dunn, "whose friendship and advice have always been of the best to me, both in connection with the Institution and my private affairs," should, as far as possible, succeed him in the position he occupied in connexion with Lord Mayor Treloar's Cripples'

Hospital and College at Alton, Hants, "he having been so closely and intimately connected with me in its work," and failing him, then that his adopted daughter, Miss Florence Treloar, should so succeed.

### Examinations for Health Workers.

At an Examination for Health Visitors and School Nurses, held at Manchester by the Royal Sanitary Institute, there were 5 candidates and the following 2 were awarded certificates: Dorothy Atkinson and Edith Muriel Hobdey. At an Examination for Sanitary Inspectors, held at Manchester, 27 candidates presented themselves, and the following 9 candidates were certified as competent to discharge the duties of Sanitary Inspector under the Public Health Act, 1875: Kenneth Hodgson Docton, Harold Downes, Frank Hewitt, William Charles McGlynn, Henry Edgar Moses, William Neville Proffitt, Richard Riley, Bernard Edgar Shaw, Norman Smith.

### The Panel Court of Inquiry.

The Court of Inquiry to be appointed by the Minister of Health and the Secretary for Scotland with reference to the amount of the capitation fee to be paid as from January 1, 1924, to practitioners serving under the National Health Insurance scheme, will be composed as follows:—Chairman, Mr. T. R. Hughes, K.C. (Chairman of the General Council of the Bar), with Mr. F. C. Goodenough (chairman of Barclays Bank) and Sir Josiah Stamp, K.B.E. It will be remembered that Sir Josiah Stamp has special knowledge of this question, having been one of the arbitrators appointed by the Government to deal with it in 1920. The terms of reference to the Committee are under consideration and will be published later.

### "Ultimate Fusion."

At a Joint Conference between the National Council for Combating Venereal Diseases and the Society for the Prevention of Venereal Disease, the following resolutions were adopted: "That the ultimate fusion of the two Societies is desirable. Subject to the approval of their respective Executives, this Conference resolves itself into a deputation to the Ministry of Health to urge that the law should be altered so as to permit properly qualified chemists to sell *ad hoc* disinfectants, provided such disinfectants are sold in a form approved and with instructions for use approved by some competent authority. That this Conference recommends that the respective Executives of the two Societies should each appoint not more than five members to form a *liaison* Committee between the two Societies and to explore the possibility of fusion."

### Health Week at Middleton.

A successful Health Week has recently been held at Middleton, organised by the M.O.H., Dr. Beggs, and the local Health Committee. Perhaps the greatest attraction was the exhibition of all manner of hygienic labour-saving appliances, including the most modern gas cookers, gas and electric lights, electric radiators and electric irons. Children from St. Michael's Schools, Tonge, had during school hours made hygienic and warm wool garments for babies, which were sold to necessitous mothers at cost price. There was also an optician's display including the testing of the eyesight and various types of spectacles or eye-glasses. A well-known firm of manufacturing chemists had a stall filled with various up-to-date surgical appliances. Each day demonstrations on these and innumerable other exhibits were given, and there were also lectures given on such subjects as "Food in Sickness" and "Food in Health," together with demonstrations on them.

### Conference on Pasteurization of Milk.

A National Milk Conference, convened by the National Clean Milk Society, to consider the question of pasteurization, was held at the Guildhall on November 21. Dr. Seligman said that, although the knowledge of milk had greatly increased, there had been no advance in the principles of its preservation since Pasteur's day. Lord Dawson of Penn deplored the small consumption of milk per head in this country. The quantity consumed per head per day was approximately a quarter of a pint, while in the United States it was at least three times as great. Dr. Beattie, Professor of Bacteriology in the University of Liverpool, was of opinion that pasteurization, properly carried out at a constant temperature rather higher than that at present in use, would bring about the destruction of all important disease-producing organisms.

### The Artificial Leg.

A pamphlet of interest to ex-service men or any who have lost a leg is issued by Captain Henry Baird, D.S.O., the late Representative of the Ministry of Pensions for the Home Counties (South). This pamphlet explains the benefits of the New Desoutter Light Metal Limb recently adopted by the Ministry of Pensions, and outlines the conditions under which the limb is supplied. Wearers of this limb have been able to ascend Snowdon and walk from London to Brighton, and Captain Baird, himself a wearer, is a well-known golfer. He will gladly supply the pamphlet on receipt of two 1d. stamps to cover postage and his address is Bridge, Canterbury.

### People's League of Health.

On the eve of his retirement from office as Lord Mayor, Sir Edward Moore wrote to enlist financial support for the Endowment Fund of the People's League of Health. "The purpose of the League, which was founded in 1917 by Miss Olga Nethersole, is to multiply and supplement the sources of public information and education to such an extent that no mother shall lose her child through ignorance: that no man or woman shall be stricken with disease for lack of knowledge. Recognising the national work that the League has undertaken in the interests of the people through the Trades Union General Council, the National Union of Teachers and the Prisons and Borstal Institutions of the country, and as Honorary Treasurer to the Endowment Fund of the People's League of Health—to which the King has graciously contributed £100—I earnestly hope that the community will give a cordial response to this appeal which, as an official member of the League, it is my privilege to make." Cheques can be sent to the Lord Mayor at the Mansion House and crossed "People's League of Health Endowment Fund."

